# Clinical implications of prospective genomic profiling of metastatic breast cancer patients

**DOI:** 10.1186/s13058-020-01328-0

**Published:** 2020-08-18

**Authors:** Courtney T. van Geelen, Peter Savas, Zhi Ling Teo, Stephen J. Luen, Chen-Fang Weng, Yi-An Ko, Keilly S. Kuykhoven, Franco Caramia, Roberto Salgado, Prudence A. Francis, Sarah-Jane Dawson, Stephen B. Fox, Andrew Fellowes, Sherene Loi

**Affiliations:** 1 Division of Research, Peter MacCallum Cancer Centre, Melbourne, Australia; 2 Department of Medical Oncology, Peter MacCallum Cancer Centre, Melbourne, Australia; 3 Australian Centre for Disease Preparedness, Commonwealth Scientific and Industrial Research Organisation (CSIRO) Health and Biosecurity, Geelong, Australia; 4 Department of Pathology, Peter MacCallum Cancer Centre, Melbourne, Australia; 5 Sir Peter MacCallum Department of Oncology, University of Melbourne, 305 Grattan St, Melbourne, VIC 3000 Australia

**Keywords:** Breast cancer, Metastasis, Genomic profiling, Personalised medicine

## Abstract

**Background:**

Metastatic breast cancer remains incurable. Next-generation sequencing (NGS) offers the ability to identify actionable genomic alterations in tumours which may then be matched with targeted therapies, but the implementation and utility of this approach is not well defined for patients with metastatic breast cancer.

**Methods:**

We recruited patients with advanced breast cancer of any subtype for prospective targeted NGS of their most recent tumour samples, using a panel of 108 breast cancer-specific genes. Genes were classified as actionable or non-actionable using the European Society of Medical Oncology Scale for Clinical Actionability of Molecular Targets (ESCAT) guidelines.

**Results:**

Between February 2014 and May 2019, 322 patients were enrolled onto the study, with 72% (*n* = 234) of patients successfully sequenced (*n* = 357 samples). The majority (74%, *n* = 171) of sequenced patients were found to carry a potentially actionable alteration, the most common being a *PIK3CA* mutation. Forty-three percent (*n* = 74) of patients with actionable alterations were referred for a clinical trial or referred for confirmatory germline testing or had a change in therapy outside of clinical trials. We found alterations in *AKT1*, *BRCA2*, *CHEK2, ESR1*, *FGFR1*, *KMT2C*, *NCOR1*, *PIK3CA* and *TSC2* to be significantly enriched in our metastatic population compared with primary breast cancers. Concordance between primary and metastatic samples for key driver genes (*TP53*, *ERBB2* amplification) was > 75%. Additionally, we found that patients with a higher number of mutations had a significantly worse overall survival.

**Conclusion:**

Genomic profiling of patients with metastatic breast cancer can have clinical implications and should be considered in all suitable patients.

## Background

Breast cancer remains the most common malignancy and cause of cancer-associated death amongst women worldwide [1, 2]. For patients with metastatic breast cancer (MBC) the disease is almost always fatal, with 5-year survival rates of approximately 26% [3, 4]. In early-stage breast cancer, treatment decisions are guided by the clinical subtypes of oestrogen receptor positive (ER+HER2−), human epidermal growth factor receptor 2 amplified (HER2+) and triple-negative breast cancer (TNBC), which are defined by the presence or absence of the oestrogen receptor and progesterone receptor and HER2 overexpression or gene amplification. This broad classification does not account for significant tumour evolution during disease progression, driven by selective pressures [1, 5]. Additionally, germline mutation testing for breast cancer patients, which could identify targetable alterations across all patients, is only common in the cases of young women, male breast cancer, or individuals with a strong family history [6].

There are an increasing number of agents that target specific genomic alterations, especially mutated protein kinases [7, 8]. Identification of suitable patients and gaining access to the corresponding targeted therapy has been challenging due to the cost of sequencing technology, the requirement in the past for non-formalin-fixed material, the inherent intra-tumoural heterogeneity of breast cancer and the lack of frequently occurring genomic alterations that can be targeted. In MBC, few genes are mutated at a frequency above 10%, leaving a wide array of infrequently altered potential targets [8]. Due to the rarity of these targets, clinical trials of targeted therapies are challenging to conduct.

In the era of precision oncology, few large-scale genomic studies have focused on metastatic breast cancer, and hence, our understanding of the breast cancer genome has relied on large-scale studies in primary tumours [7, 9, 10]. However, it is now well established that early breast cancer and MBC may have differing genomic profiles even in the same patient. More data surrounding the genomic landscape of MBC will be useful to identify oncogenic drivers specific for advanced disease with potential therapeutic implications, such as *ESR1* and resistance to endocrine therapies [10, 11].

Here we describe such an initiative, from an academic tertiary referral cancer centre, where individuals with MBC had their tumour samples prospectively profiled using a targeted next generation sequencing (NGS) assay. The aim of this study was to explore the feasibility and clinical relevance of this approach.

## Methods

### Patient recruitment and samples

From January 2014 to May 2019, 322 patients were recruited from across Australia. All patients signed informed consent at Peter MacCallum Cancer Centre outpatient clinics (ethics committee approved study 13/123). Key inclusion criteria included age 18 years or over, histological confirmation of breast cancer and a life expectancy greater than 3 months. Patients of any gender, and with any breast cancer subtype or sites of disease, were allowed on study. Following successful enrolment, baseline information including relevant medical history, Eastern Cooperative Oncology Group performance status, past treatment and intended treatment were collected. Updates to the clinical history including survival follow-up were evaluated every 3 months.

Tumour tissue was requested for all recruited patients from various anatomical pathology service providers. Wherever possible, both primary and metastatic samples were obtained. Tissues were received as formalin-fixed, paraffin-embedded (FFPE) unstained sections at 5-μM thickness with up to 20 sections requested per sample. Prior to DNA extraction, a single slide was stained with haematoxylin and eosin to confirm the presence of cancer and to evaluate for tumour cellularity and tumour-infiltrating lymphocytes (TIL) in the sample [12].

### Next-generation sequencing genomic profiling

Tumour DNA was extracted after micro- or macro-dissection (sample dependent) on sections stained with methyl green and quantified using the Fluorometric Qubit High Sensitivity system (ThermoFisher Scientific, USA). A minimum of 100 ng of DNA was required to proceed to sequencing.

A custom gene panel targeting all exons of genes recurrently altered in breast cancer was designed for the Agilent SureSelectXT Target Enrichment System (Agilent Technologies, Santa Clara, CA, USA) using the SureDesign tool (Table 1). This platform utilises biotinylated RNA baits for hybrid capture of desired DNA. Prior to library preparation with the KAPA Hyper Prep Kit (KAPA Biosystems, USA), DNA for each sample was randomly fragmented using ultrasonication (Covaris Inc., USA). Libraries passing quality control underwent hybrid capture with the custom RNA baits using the Bravo automated liquid handling robot (Agilent Technologies, Australia). Captured libraries were indexed and pooled prior to paired end sequencing on the Illumina MiSeq or NextSeq. A process matched non-tumour control sample was included in each run for use in downstream analysis. A total of 20 runs have been performed over the study, with a range of 10–40 samples per run.

**Table 1:** Targeted sequencing panel and actionable genes as per ESMO guidelines

Gene	Alteration type	
**LEVEL 1: “known actionable alteration”**		
*AKT1*	E17K, other pathogenic mutations	
*BRCA1/ BRCA2*	Germline mutations	
*ESR1*	Somatic mutations, previously reported	
*ERBB2*	Amplification and mutation	
*PIK3CA*	Somatic mutations	
*PTEN*	Homozygous deletions, loss of function mutations	
**LEVEL 2: “potentially actionable alteration”**		
*ARID1A*	Somatic mutations	
*ATM*	Somatic mutations	
*BRCA1/BRCA2*	Somatic mutations	
*CDH1*	Somatic mutations	
*ERBB3*	Somatic mutations	
*HRAS*	Somatic mutations	
*IGF1R*	Somatic mutations	
*INPP4B*	Somatic mutations	
*MAP2K4*	Somatic mutations	
*MAP3K1*	Somatic mutations	
*MDM2*	Amplifications	
*PIK3R1*	Somatic mutations	
*RB1*	Somatic mutations	
**Non-actionable genes**		
*AKT2*	*FANCC*	*NRAS*
*AKT3*	*FANCG*	*PALB2*
*ALK*	*FBXW7*	*PDGFRA*
*APC*	*FGFR1*	*PIK3R3*
*AXIN2*	*FGFR2*	*PMS2*
*BAP1*	*FGFR3*	*PRKAR1A*
*BLM*	*FGFR4*	*PTCH1*
*BMPR1A*	*FH*	*PTPN11*
*BRAF*	*FLCN*	*RAD51C*
*BRIP1*	*FOXA1*	*RAD51D*
*BUB1B*	*GATA3*	*RECQL4*
*CASP8*	*KIT*	*RET*
*CBFB*	*KRAS*	*RUNX1*
*CCND1*	*MAP2K1*	*SDHA*
*CCND2*	*MAX*	*SDHB*
*CDC73*	*MCL1*	*SDHC*
*CDK4*	*MEN1*	*SDHD*
*CDKN2A*	*MET*	*SF3B1*
*CHEK2*	*MLH1*	*SMO*
*CTNNB1*	*KMT2D*	*STK11*
*DDB2*	*KMT2C*	*TBX3*
*EGFR*	*MSH2*	*TMEM127*
*ERBB4*	*MSH6*	*TP53*
*ERCC2*	*MUTYH*	*TSC1*
*ERCC3*	*NBN*	*TSC2*
*ERCC4*	*NCOR1*	*VHL*
*ERCC5*	*NF2*	*WT1*
*EXT1*	*NOTCH1*	*XPA*
*FANCA*	*NOTCH2*	*XPC*

Level 1 actionable alterations are equivalent to ESMO ESCAT LOE I and II, whilst level 2 actionable alterations are equivalent to LOE III and IV as described in [13]

### Assessment of gene actionability

Table 1 is adapted from the European Society for Medical Oncology (ESMO) and their European Society of Medical Oncology Scale for Clinical Actionability of Molecular Targets (ESCAT) guidelines [13]. These classify the actionability of genes according to various levels of evidence (LOE). As indicated in Table 1, level 1 actionable corresponds to ESCAT LOE I/II, whilst level 2 actionable genes is equivalent to LOE III/IV. Although amplification of *ERBB2* is considered to be a level 1 actionable alteration, because it is the defining factor of the HER2+ breast cancer subtype, it is not included in the analysis. All patients classified as HER2+ would therefore have a level 1 actionable alteration; it is excluded from the analysis because it defines the HER2+ breast cancer subtype and is part of standard diagnostic testing.

### Tumour-infiltrating lymphocyte (TIL) quantity evaluation

TIL analysis was performed wherever possible on available H&E slides as per the International TILs Working Group guidelines by a single pathologist (RS) [14].

### Bioinformatics analysis

Raw sequencing data had adapters trimmed with cutadapt (v1.6–1.9.1) [15]. The Burrows-Wheeler Aligner algorithm was used to align sequencing reads to the human genome (GRCh37), with no altered parameters [16]. The Depth of Coverage tool in the Genome Analysis Tool Kit (GATK 3) was used to calculate the mean coverage accounting for overlapping read pair ends [17]. Duplicate reads were removed with Picard Tools (v1.77–v1.141) [18]. VarDict (v1.3.0–1.5.7) was used to call variants that were then annotated with ANNOVAR [19, 20]. CopywriteR (v2.0.1–2.0.6) was used to call copy number (CN) alterations from off-target reads with a bin width of 50 kb [21]. A mean log ratio > 0.7 was considered amplified, and < 0.7 indicative of a heterozygous or homozygous deletion.

Manual curation was then used to identify the variants most likely pathogenic and relevant to the patient’s disease. This included removing samples with a read depth of less than 100, a variant allele frequency less than 5% and variants with strand bias. Additionally, only variants that led to a protein change (non-synonymous), those located in the exonic region of the gene and those not found in either the 1000 genomes population or the ESP650 cohorts were included in the analysis [22, 23]. Integrative Genomics Viewer was then utilised to confirm the presence of the mutation and exclude sequencing error or variants in highly repetitive regions (either homopolymers > 4 bases in length or triplet nucleotide repeats) [24]. Any mutation in *TP53*, *BRCA1*, or *BRCA2* that had a variant allele frequency greater than 20% was referred for consideration of germline testing.

A report was generated on the sequencing results of each patient, including an estimation of the likelihood of the mutation’s oncogenic relevance and potential therapeutic options including clinical trials. This estimation of oncogenic relevance was calculated using various resources including the ClinVar, the Catalogue of Somatic Mutations in Cancer, OncoKB and medical literature [25–27].

### Statistical analysis

Survival analysis was performed using Cox regression models. In all cases, overall survival was calculated from date of study consent to either date of death or date of last follow-up. Time of diagnosis for both primary and metastatic disease was calculated from the first biopsy confirmation of cancer, or if a biopsy was unsuitable, first imaging to confirm the presence of cancer. Somatic interaction analysis and calculation was performed using the pair-wise Fisher’s exact test to detect co-occurring gene pairs and the CoMet ExactTest algorithm [28–30]. Concordance scores were calculated by dividing the number of concordant observations by the total number of observations per genetic alteration. The same principle was applied to calculate individual patient concordance scores. Calculations for mutation numbers were performed using one sample from every patient, ideally a metastatic sample; however, when no metastatic sample was available, a primary sample was included instead. Adjustment for multiple testing was performed using the method of Benjamini and Hochberg. All statistical analysis was performed using R software (R version 3.6.2 (2019-12-12)).

## Results

Between February 2014 and May 2019, 322 patients with MBC were recruited. The majority of these patients were ER+HER2− (68%, *n* = 219), with HER2+ and TNBC representing 12% (*n* = 38) and 20% (*n* = 65) respectively. The median age of these patients was 46 years (23–74) at primary diagnosis and 52 years (24–78) at metastatic diagnosis (Table 2).

**Table 2:** Study cohort clinical features

	Overall (***n*** = 322)	ER+HER2− (***n*** = 219, 68%)	HER2+ (***n*** = 38, 12%)	TNBC (***n*** = 65, 20%)	***P*** value
Median age at primary diagnosis (range)	46 (23–74)	46 (24–73)	43.5 (34–74)	47 (23–74)	
Median age at metastatic diagnosis (range)	51 (24–78)	52 (30–78)	45.5 (35–67)	50 (24–76)	
Median age at consent (range)	52 (24–81)	54 (30–78)	48 (36–81)	52 (24–77)	
Median DFI^1^ (range)	40 (1–312)	54.5 (1–312)	25 (4–136)	23 (1–252)	
De novo patients	71 (22%)	50 (22%)	14 (36%)	7 (11%)	
**Treatment history**					
**Previous chemotherapy**					
Yes	309 (96%)	208 (95%)	37 (97%)	64 (98%)	
No	8 (2%)	7 (3%)	1 (3%)	0	
Unknown	5 (2%)	4 (2%)	0	1 (2%)	
**Previous Endocrine therapy**					Chi squared: < 0.0001
Yes	222 (69%)	205 (94%)	11 (29%)	6 (9%)	
No	95 (29%)	10 (5%)	27 (71%)	58 (89%)	
Unknown	5 (2%)	4 (1%)	0	1 (2%)	
**Lines of treatment in metastatic setting**					Chi squared: 0.003
1	28 (9%)	12 (5%)	1 (3%)	16 (25%)	
2	70 (22%)	47 (21%)	4 (11%)	19 (29%)	
3	72 (22%)	45 (21%)	13 (34%)	14 (22%)	
> 3	133 (41%)	103 (47%)	20 (53%)	10 (15%)	
Unknown	20 (6%)	12 (5%)	0	6 (9%)	
**Sequenced patients**					
Sequenced	234 (72%)	162 (75%)	25 (66%)	47 (72%)	
Not sequenced due to sample failure	18 (6%)	12 (6%)	1 (3%)	5 (8%)	
Not sequenced due to other^2^	70 (22%)	45 (19%)	12 (31%)	13 (20%)	
**Patients with actionable mutations**^**3**^	171 (74%)	131 (80%)	11 (44%)	29 (61%)	Chi squared: 0.0001
Level 1:	114 (49%)	93 (57%)	6 (24%)	15 (32%)	
Level 2	57 (25%)	38 (24%)	5 (20%)	14 (30%)	
No actionable alterations	63 (26%)	31 (19%)	14 (56%)	18 (38%)	

*DFI* disease-free interval (time in months)

^1^Patients with de novo metastatic disease not included. ^2^Other reasons for not being sequenced aside from sequencing failure include: sample not received or insufficient DNA quantity for sequencing. ^3^Percentage calculated from number of sequenced patients. Numbers shown are reflective of the overall total and then the total within each subtype. The HER2+ subtype does not include patients with *ERBB2* amplification (100%, *n* = 25)

**Fig. 1:**
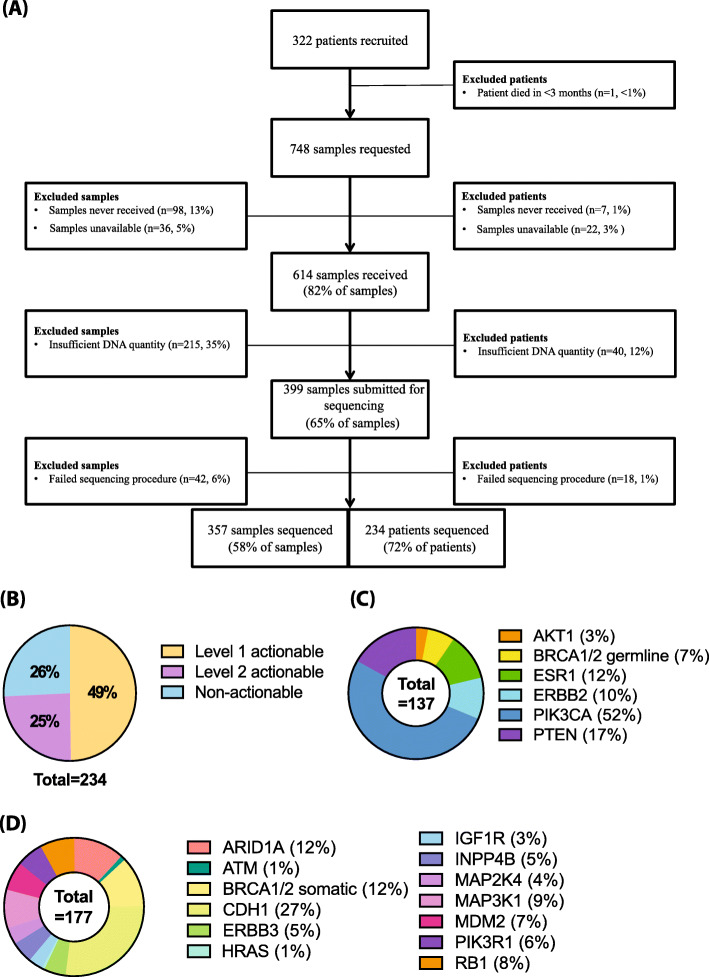
Study cohort. **a** Recruitment and specimen retrieval flow chart. Sample information and numbers are shown on the left of the central chart and patient information and numbers are shown on the right. **b** Actionable alterations. Pie chart shows the percentage breakdown of patients and the level of actionable alterations their samples contain. Patients with at least one level 1 actionable alteration amounted to 49% (*n* = 114). Patients with a level 2 actionable alteration accounted for 25% (*n* = 57). Patients without an actionable alteration amounted to 26% of the cohort (*n* = 60). **c** Level 1 actionable alteration breakdown by number of samples. *AKT1* mutations (3%, *n* = 4), *BRCA1/2* germline variants (7%, *n* = 9), *ESR1* mutations (12%, *n* = 16), *ERBB2* mutations (10%, *n* = 14), *PIK3CA* mutations (52%, *n* = 71) and *PTEN* mutations (17%, *n* = 23). *ERBB2* amplifications are not included. **d** Level 2 actionable alteration breakdown by number of samples. *ARID1A* mutations (12%, *n* = 21), *ATM* mutations (1%, *n* = 2), *BRCA1/2* non-germline mutations (12%, *n* = 21), *CDH1* mutations (27%, *n* = 48), *ERBB3* mutations (5%, *n* = 9), *HRAS* mutations (1%, *n* = 1), *IGF1R* mutations (3%, *n* = 6), *INPP4B* mutations (5%, *n* = 9), *MAP2K4* mutations (4%, *n* = 7), *MAP3K1* mutations (9%, *n* = 16), *MDM2* amplifications (7%, *n* = 12), *PIK3R1* mutations (6%, *n* = 11) and *RB1* mutations (8%, *n* = 14)

Across the cohort, 748 tumour samples were requested, with an average of 2 samples per patient (Fig. 1a). In total, 82% (*n* = 614) of the requested samples were received; however, the time between request and receipt varied considerably with an average of 29 days for samples stored by the parent institution and 49 days for samples retrieved from external pathology providers.

Amongst the received samples, 35% (*n* = 215) had insufficient DNA quantity for sequencing, and the remaining 65% (*n* = 399) underwent sequencing with a success rate of 90% (*n* = 357). The failure rate of 10% of samples (*n* = 42) was largely attributed to poor quality DNA due to FFPE fixation, as neither the age of the sample nor its location were significant predictors of failure. The majority of the failed sequencing samples came from primary lesions (70%, *n* = 29). Metastatic samples, from sites such as lymph node (12%, *n* = 5), liver (5%, *n* = 2) and lung (5%, *n* = 2), accounted for less than a quarter of sequencing failures. A single specimen from the bone, ovary, brain and skin also failed sequencing (2% each). Ultimately, this led to 72% (*n* = 234) of recruited patients being successfully sequenced. The majority of samples sequenced successfully were primary tumours (53%, *n* = 189). Samples from metastatic lymph nodes (10%, *n* = 37) and the liver (9%, *n* = 31) accounted for the majority of metastatic samples (*n* = 168, 47%). Other metastatic sites included bone (7%, *n* = 26), brain (5%, *n* = 19), lung (3%, *n* = 12) and ovarian (2%, *n* = 6). The remaining 6 metastatic samples each came from a unique site and accounted for overall 2% (*n* = 6). Paired primary and metastatic tissue was available for 35% of patients (*n* = 81).

Across the sequenced samples, there was a median of 3 non-synonymous somatic mutations per sample (range 0–10) and 4 focal CN alterations (range 1–28). Level 1 alterations were found in 49% (*n* = 114) of all patients sequenced and 25% (*n* = 57) of all patients were found to carry a level 2 change (Fig. 1b). This amounts to 74% (*n* = 171) of patients harbouring a potentially actionable alteration. The majority of these samples were of the ER+HER2− subtype (77%, *n* = 131), with HER2+ and TNBC subtypes accounting for 6% (*n* = 11) and 17% (*n* = 29) of the total cohort respectively. Fifty-seven percent of ER+HER2− cases (*n* = 93/162), 24% of HER2+ (*n* = 6/25) and 32% of TNBC cases (*n* = 15/47) had level 1 alterations.

As shown in Fig. 1c, *PIK3CA* was the most common level 1 actionable alteration, being found in 30% of sequenced patients and accounting for 52% of patients with level 1 actionable mutations (*n* = 71). Of the cohort with actionable alterations (*n* = 171), 43% (*n* = 74) were referred onto a molecularly targeted clinical trial. The majority of these patients had *PIK3CA* mutations (89%, *n* = 68), followed by 2 patients with *PTEN* mutations, and 4 patients with *ERBB2* mutations. A further 2 patients with *FGFR1* amplifications were referred for FGFR inhibitor trials, although this gene is not considered actionable according to ESMO Clinical Actionability guidelines [13]. Of ER+HER2− patients, 10% (*n* = 24) were found to carry an *ESR1* mutation in their metastatic tumour sample. Nearly all of these patients had been treated with prior endocrine therapy (Table 2).

A total of 21 patients were found to harbour *BRCA1* or *BRCA2* mutations. Of these, 36% (*n* = 8) were previously known pathogenic germline *BRCA1* or *BRCA2* carriers. The remaining 64% (*n* = 13) were referred on to germline testing. Of these patients, 2 were confirmed to carry germline variants of unknown significance, 5 confirmed wild type and 6 patients did not attend germline testing. A previously known pathogenic germline mutation in *CHEK2* was also identified in a single patient.

**Fig. 2:**
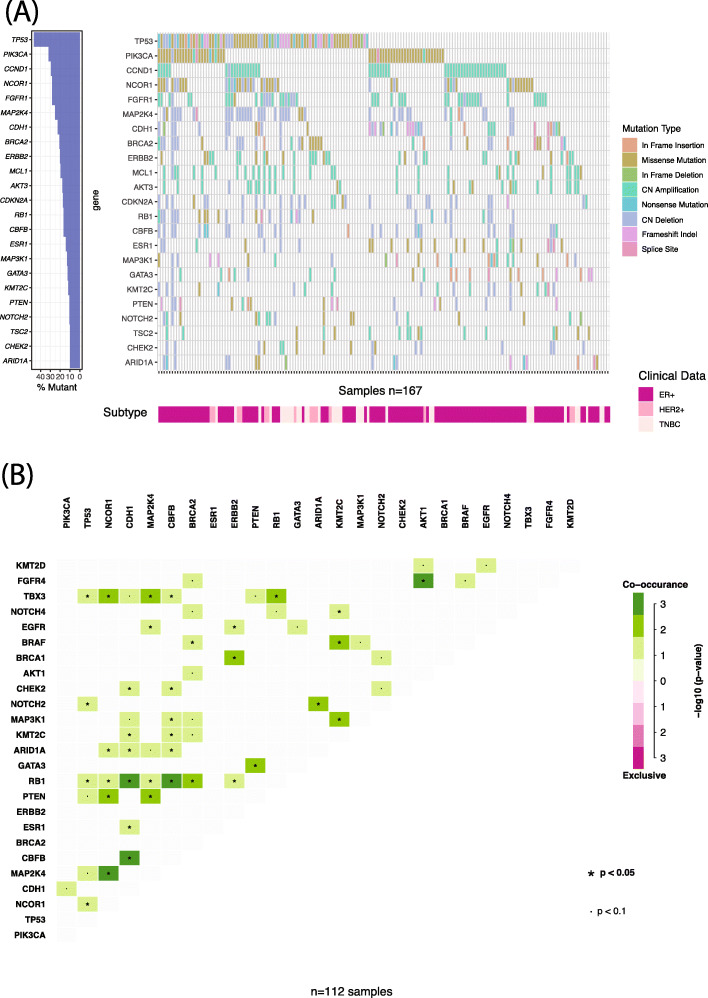
Metastatic mutational landscape. **a** Oncoplot of somatic mutations identified at a frequency of 10% or more in sequenced metastatic samples (*n* = 168). Percentage frequency of the genes is shown in the barchart to the left of the central plot. Clinical subtype and mutation type are indicated on their respective legend. For genes significantly enriched in this cohort compared to TCGA, see Supplementary Table 1. **b** Mutational co-occurrence plot for metastatic ER+HER2− samples (*n* = 112). Degree of significance indicated in the legend, with only results of the top 25 genes shown. No mutational exclusivity pairs were identified. For individual *p* values, see Supplementary Table 2

Figure 2a shows the genomic landscape of the sequenced metastatic samples (*n* = 167 samples). Amongst these samples, *TP53* was the most commonly altered at 40%, followed by *PIK3CA*, *CCND1*, *NCOR1* and *FGFR1*. In order to identify any gene that may be enriched in the metastatic cohort, the mutational frequencies were compared to those in the TCGA breast cancer dataset comprised of primary tumours [31]. The genes *AKT1*, *BRCA2*, *CHEK2*, *ESR1*, *FGFR4*, *KMT2C*, *NCOR1*, *PIK3CA* and *TSC2* were significantly enriched in our metastatic study population compared to the TCGA cohort (Supplementary Table 1).

We performed an analysis to identify alterations that may be significantly co-occurring or mutually exclusive. Each subtype was analysed individually using the 25 most frequently altered genes within each cohort. In ER+HER2− metastatic samples (*n* = 112) (Fig. 2b and Supplementary Table 2), 43 gene-pairs were found to significantly co-occur. When this analysis was performed in the TNBC subtype (*n* = 40 samples), only 2 gene pairs were found to be co-occurring: *RB1* and *FGFR1* (*p* = 0.01), as well as *CHEK2* and *CDH1* (*p* = 0.025). In the ER+HER2− group, *RB1* mutations were found to co-occur with 8 other genes, suggesting a significant role within this subtype (Supplementary Table 2). Overall, in our cohort TNBC metastatic samples have less significantly co-occurring genes than ER+HER2− breast cancers.

**Fig. 3:**
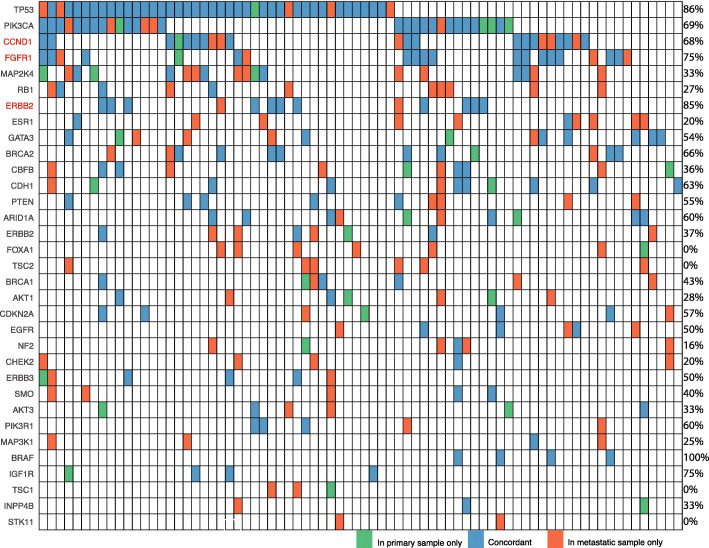
Concordance plot of primary and metastatic paired samples. Data for 76 patients and their paired sequenced samples is shown. Genetic alterations included in this figure are putative driver genes observed in 2 or more samples. A further 4 patients and their paired samples have been excluded from the figure because their pairs did not include the genes shown. Concordance scores for each gene is listed on the right of the figure. Genes listed in red indicate that it is CN amplification concordance being compared, rather than mutation concordance (shown in black text)

The concordance of key driver alterations was investigated between primary and metastatic samples (Fig. 3) across the 81 patients (35%) of the cohort with paired samples. Of note, *PIK3CA* was largely concordant with a score of 69% (*n* = 20/29 patients). In the 9 patients who displayed discordance, the *PIK3CA* mutation was found exclusively in the metastatic lesion in 5 patients and exclusively in the primary lesion in 4 patients. The only gene to show 100% concordance was *BRAF* (*n* = 4 patients); however, *TP53*, *FGFR1* amplification, *ERBB2* amplification and *IFG1R* showed concordance scores greater than 75%. *FOXA1*, *STK11*, *TSC1* and *TSC2* were found to have no concordance between tumours and were largely restricted to metastases (Fig. 3). When tumour concordance was evaluated within each patient, only 18% (*n* = 15 patients) were found to have 100% concordance between their primary and metastatic lesion, whilst 7% of patients (*n* = 6) had no concordant findings, highlighting the genomic heterogeneity of metastatic breast cancer within individual patients.

**Fig. 4:**
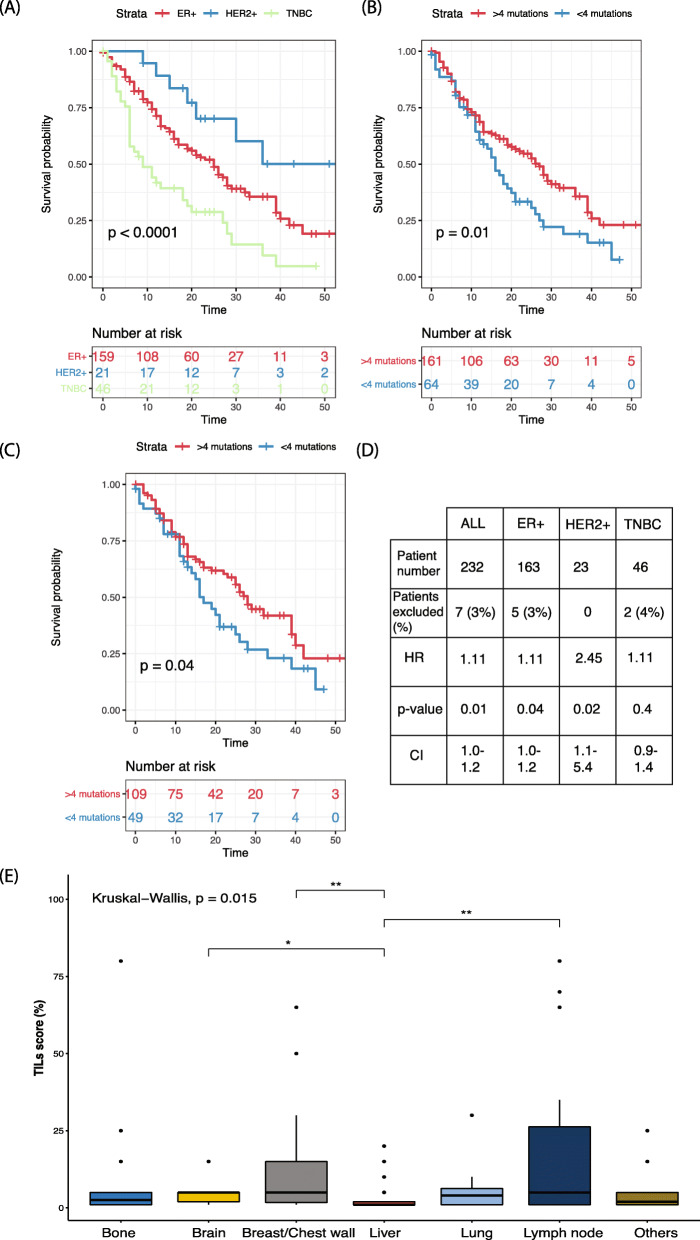
Prognostic associations in this cohort of sequenced metastatic breast cancer patients. **a** Overall survival by subtype for all recruited patients (*n* = 323). **b** Overall survival of patients based on the mutational burden of 4 mutations (75th percentile) or more (*n* = 234). **c** Overall survival of ER+HER2− patients based on the median mutation number of 4 or more (*n* = 163 patients). **d** Table of HR for all patients and all subtypes by mutation number per sample. Patients were excluded if there was incomplete survival information. **e** Spread of TILs across distant metastatic site (*n* = 123)

We investigated factors associated with survival, and unsurprisingly, the clinical subtype (Fig. 4a) had a significant impact on overall survival (OS). When the time between primary disease and metastatic disease diagnosis was compared amongst the patients, those with a longer disease-free interval had a significantly better OS (hazard ratio (HR) = 0.61, confidence interval (CI) = 0.39–0.94, *p* = 0.02). Amongst the patients with sequenced samples, we found that higher mutation number was associated with significantly worse survival (HR = 1.1 per mutation, CI = 1.0–1.2, *p* = 0.01, Fig. 4b). This trend was observed in both the ER+HER2− subtype (HR 1.1, CI = 1.0–1.2, *p* = 0.04; Fig. 4c) and the HER2+ subtype (HR = 2.5, CI = 1.1–5.3, *p* = 0.02), but not in the TNBC subtype (HR = 1.1, CI = 0.86–1.4, *p* = 0.4; Fig. 4d). The same subtype level analysis was performed using copy number; however, no significant results were observed.

Although breast cancer is not commonly considered to be immunogenic, the presence of TILs has been known to impact survival in primary breast cancer [32]. In our dataset, TILs in the metastatic lesion were not significantly associated with OS for any subtype, as calculated from time of metastatic diagnosis (Supplementary Table 3). As anticipated, amongst the primary samples, TNBC patients with a higher number of TILs had a significantly longer DFI (Supplementary Table 3). This was also evident using the TNBC metastatic samples, where those with higher TILs had a significantly longer disease-free interval than those with lower TILs (HR 4.07, CI 1.2–13.6, *p* value: 0.02). Consistent with previous data some metastatic sites had significantly greater TIL infiltrate (Fig. 4e) [33].

## Discussion

In this study, 49% (*n* = 114) of patients were found to harbour a level 1 actionable alteration, which corresponds to the ESCAT LOE I/II genes. Level 1 actionable alterations were of high clinical relevance with 65% (*n* = 74/111) of patients with alterations in this category referred for clinical trial. Level 2 actionable alterations were identified in 25% (*n* = 57) of patients and did not impact clinical decision making due to the unavailability of a matched therapy. This amounts to almost three-quarters (74%, *n* = 171) of the cohort being found to harbour at least one actionable alteration, albeit with variable clinical importance. Of ER+HER2− patients, 10% (*n* = 24) were found to carry *ESR1* alterations, and 64% (*n* = 13) of patients with a *BRCA1* or *BRCA2* mutation with a high variant allele fraction were recommended for germline testing. Additionally, we found several genes that were enriched in the metastatic setting across the whole cohort, as well as discordant findings in patients with both primary and metastatic samples, highlighting the need to profile metastatic tissue where possible. Finally, we found that higher mutation number was also associated with worse survival. These findings were obtained using a targeted panel sequencing far fewer genes (around one-third) than some which are commercially available. The outcomes of this study suggest that a panel featuring only ESCAT LOE I/II genes, equivalent to level 1 in this study, would be sufficient for the majority of patients who could benefit from genomic interrogation. These smaller and less expensive assays may make genomic profiling more broadly accessible to breast cancer patients and clinicians.

The distribution and frequency of metastatic genomic alterations in this study are similar to the findings of other studies, including enrichment of *ESR1* mutations believed to be the result of therapeutic selective pressure [7, 10, 11, 34]. Mutations in *ESR1* are almost exclusively found in metastatic lesions of ER+HER2− patients, although some studies have shown they can exist prior to the onset of metastases in patients treated with endocrine therapies [35, 36]. This was observed in 2 patients, shown in Fig. 3, where the primary samples were collected post-endocrine therapy and had already developed an *ESR1* alteration. *ESR1* mutations arise due to prolonged treatment with tamoxifen and aromatase inhibitors; however, retrospective analysis of the PALOMA-2 and PALOMA-3 studies found that patients treated with a selective oestrogen degrader either alone or in combination with CDK4/6 inhibitors also showed development of *ESR1* mutations [37–39].

As expected, *TP53* alterations were the most prevalent across all samples. Of all the level 1 actionable genes, *PIK3CA* was found to be the most commonly altered, consistent with other studies [34]. Recently, the α-subunit specific *PIK3CA* inhibitor alpelisib was approved for use in ER+HER2− MBC patients with hotspot *PIK3CA* mutations, providing a matched therapy for this most common alteration [34, 40, 41]. Conversely, the finding that 25% of patients carried a level 2 alteration which lack proven therapeutic options highlights the difficulties in matching the majority of patients with targeted therapies. It is uncertain how many of these level 2 alterations may be clinically relevant in the near future. Nevertheless, the high prevalence of *PIK3CA* alterations alone means that a nearly a quarter of MBC patients could be treated with a matched targeted therapy, even accounting for those that could not have a metastatic sample sequenced as there was a high concordance rate (69% concordance) with the primary tumour.

Like other studies, we found a proportion of patients (26%) did not carry a potentially actionable alteration. Patients where no alteration was found may benefit from more comprehensive genomic analysis such as whole-genome sequencing, where mutational signatures such as homologous recombination repair deficiency or more rarely, microsatellite instability could be identified and utilised for therapeutic decisions [42, 43].

Our concordance analysis in 81 patients with metachronous primary and metastatic samples allows observation of how key driver genes change in metastatic disease. Mutations in both *TSC2* and *STK11* were found exclusively in metastatic samples and are possibly associated with the development of metastatic disease or resistance to therapy. The finding that *TSC2* was also enriched in our metastatic cohort compared to the TCGA primary data set also supports this hypothesis [11]. The high frequency of mutations in the genes *RB1*, *TSC1* and *FOXA1* in the metastatic samples with few or no concordant cases also suggests these genes are similarly relevant to the biology of metastatic disease. Co-mutation analysis in both the ER+HER2− and TNBC subgroups suggested that *RB1* may also play a key role in the emergence of metastatic and treatment-resistant subclonal elements. This finding is supported by other studies that have linked alterations in *RB1* with resistance to endocrine therapies and CDK4 inhibitors [10, 44, 45].

Our study has several limitations. Firstly, the somatic landscape of the sequenced tumour may not be indicative of the whole metastatic disease burden. Novel methods such as circulating tumour DNA sequencing could be utilised to avoid sampling bias and increase the number of patients for whom genomic information is available. Nevertheless there are still cases of discordance between circulating tumour DNA and tumour DNA from sequenced samples, and plasma sampling has a higher rate of false negatives [1, 46]. Secondly, a panel of the size employed in this study cannot accurately estimate the true total mutation burden, and the mutation number per sample reported here cannot be considered a surrogate measure. Thirdly, the cohort of patients in our study may not be representative of the general metastatic breast cancer population as there was likely to be some bias towards enrolling younger and fitter patients. Fourthly, the rate at which patients receive therapies matched to their genomic profile is subject to the availability of these therapies and clinical trials, which will vary greatly across clinical contexts, and the timeframe in which the genomic profile can be delivered. The latter was heavily influenced by the time required to receive tissue samples. Reducing this further will require closer integration of pathology services with the treatment team and greater awareness of the urgency of tissue requests. Fifthly, consistent with other studies, we found that a relatively high rate of patients could not be delivered a genomic profile due to the difficulty in obtaining a suitable sample as 35% of samples had insufficient DNA. Due to the inherent difficulties in obtaining metastatic biopsies from MBC patients, these failure rates for tumour sequencing are unlikely to improve.

## Conclusion

This study has found that prospective genomic sequencing for the management of MBC is both feasible and of utility as an adjunct to standard management with 49% of sequenced patients carrying a level 1 actionable alteration. *PIK3CA* was the most common actionable mutation, found in 52% (*n* = 71) of patients with a level 1 actionable alteration. Of patients referred for a clinical trial with a matched therapy, 89% (*n* = 68) had a *PIK3CA* mutation. We found that *PIK3CA* mutation status, and genomic profiles in general, demonstrates within-patient heterogeneity which is clinically relevant, although *PIK3CA* was usually concordant across primary and metastatic samples. Nevertheless, the presence of genomic heterogeneity indicates sequencing of metastatic lesions is preferred if possible. Due to the inherent difficulties in obtaining biopsies of metastatic disease, the combination of tumour and circulating tumour DNA sequencing is likely to be the optimal strategy to improve the rate of successful genomic profiling. It is expected the clinical utility of genomic profiling will increase over time with greater availability of targeted therapies, as has already transpired with the recent approval of the *PIK3CA* inhibitor alpelisib. We conclude that where possible, patients with MBC should undergo molecular profiling.

## Supplementary information

**Additional file 1: Supplementary Table 1.** List of genes enriched in study dataset compared to the TCGA dataset. **Supplementary Table 2.** Genes-pairs that co-occur in ER+ metastatic breast cancer. **Supplementary Table 3.** Hazard ratios and confidence intervals for TILs samples.

## Data Availability

De-identified genomic variant data is available upon request to the corresponding author.

## Abbreviations

CI: Confidence interval
CN: Copy number
COSMIC: Catalogue of somatic mutations in cancer
DFI: Disease-free interval
ER +: Oestrogen-receptor positive
ESCAT: European Society of Medical Oncology Scale for Clinical Actionability of Molecular Targets
ESMO: European Society of Medical Oncology
FFPE: Formalin-fixed paraffin-embedded
HER2+: Human epidermal growth factor receptor 2 amplified
HR: Hazard ratio
LOE: Levels of evidence
NGS: Next generation sequencing
OS: Overall survival
TIL: Tumour-infiltrating lymphocytes
TNBC: Triple-negative breast cancer
