# Correction to: Clinical implications of prospective genomic profiling of metastatic breast cancer patients

**DOI:** 10.1186/s13058-022-01539-7

**Published:** 2022-07-15

**Authors:** Courtney T. van Geelen, Peter Savas, Zhi Ling Teo, Stephen J. Luen, Chen-Fang Weng, Yi-An Ko, Keilly S. Kuykhoven, Franco Caramia, Roberto Salgado, Prudence A. Francis, Sarah-Jane Dawson, Stephen B. Fox, Andrew Fellowes, Sherene Loi

**Affiliations:** 1 Division of Research, Peter MacCallum Cancer Centre, Melbourne, Australia; 2 Department of Medical Oncology, Peter MacCallum Cancer Centre, Melbourne, Australia; 3 Australian Centre for Disease Preparedness, Commonwealth Scientific and Industrial Research Organisation (CSIRO) Health and Biosecurity, Geelong, Australia; 4 Department of Pathology, Peter MacCallum Cancer Centre, Melbourne, Australia; 5 Sir Peter MacCallum Department of Oncology, University of Melbourne, 305 Grattan St, Melbourne, VIC 3000 Australia

## Correction to: Breast Cancer Research (2020) 22:91 10.1186/s13058-020-01328-0

Following publication of the original article [1], the authors identified some errors in Fig. 4. The correct figure is given below.

**Fig. 4:**
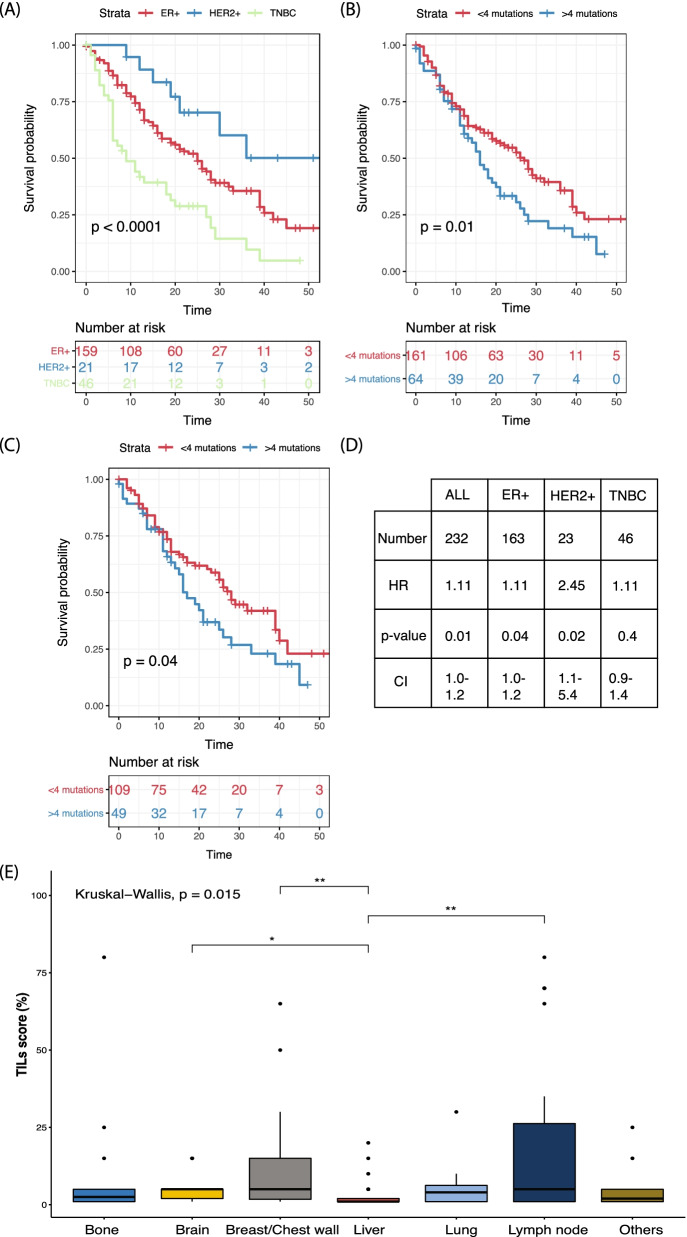
Prognostic associations in this cohort of sequenced metastatic breast cancer patients. **a** Overall survival by subtype for all recruited patients (*n* = 323). **b** Overall survival of patients based on the mutational burden of 4 mutations (75th percentile) or more (*n* = 234). **c** Overall survival of ER + HER2 − patients based on the median mutation number of 4 or more (*n* = 163 patients). **d** Table of HR for all patients and all subtypes by mutation number per sample. Patients were excluded if there was incomplete survival information. **e** Spread of TILs across distant metastatic site (*n* = 123)

The original article [1] has been corrected.
