# Supplementary material for: Clinical implications of prospective genomic profiling of metastatic breast cancer patients
Source: Breast Cancer Res. 2020 Aug 18;22:91. doi: 10.1186/s13058-020-01328-0 (PMC7436992; doi:10.1186/s13058-020-01328-0)
Supplement: Supplementary file 1 — Additional file 1: Supplementary Table 1. List of genes enriched in study dataset compared to the TCGA dataset. Supplementary Table 2. Genes-pairs that co-occur in ER+ metastatic breast cancer. Supplementary Table 3. Hazard ratios and confidence intervals for TILs samples. [file 13058_2020_1328_MOESM1_ESM.docx]

**Supplementary Table 1: List of genes enriched in study dataset compared to the TCGA dataset.**

| **Gene name** | **p-value** | **Adjusted p-value** |
| --- | --- | --- |
| AKT1 | <0.0001 | <0.0001 |
| BRCA2 | <0.0001 | <0.0001 |
| CHEK2 | 0.006 | 0.03 |
| ESR1 | <0.0001 | <0.0001 |
| FGFR4 | 0.008 | 0.03 |
| KMT2C | 0.004 | 0.02 |
| NCOR1 | <0.0001 | <0.0001 |
| PIK3CA | 0.002 | 0.02 |
| TSC2 | 0.01 | 0.05 |

Study dataset was compared to the TCGA 2018 Cancer Cell (filtered for breast cancer only), downloaded from cBioPortal.

**Supplementary Table 2: Genes-pairs that co-occur in ER+ metastatic breast cancer.**

| **Gene 1** | **Gene 2** | **p-value** | **Adjusted p-value** |
| --- | --- | --- | --- |
| CBFB | CDH1 | 5.04E-11 | >0.0001 |
| RB1 | CBFB | 4.70E-05 | >0.0001 |
| FGFR4 | AKT1 | 0.000225 | >0.0001 |
| NCOR1 | MAP2K4 | 0.00034 | >0.0001 |
| RB1 | CDH1 | 0.000904 | >0.0001 |
| BRCA1 | ERBB2 | 0.001093 | >0.0001 |
| NOTCH2 | TSC1 | 0.001358 | >0.0001 |
| TBX3 | NCOR1 | 0.001359 | >0.0001 |
| RB1 | BRCA2 | 0.001464 | >0.0001 |
| NCOR1 | PTEN | 0.001944 | >0.0001 |
| PTEN | MAP2K4 | 0.002762 | >0.0001 |
| TBX3 | MAP2K4 | 0.004474 | >0.0001 |
| ARID1A | NOTCH2 | 0.005118 | 0.02 |
| TBX3 | RB1 | 0.006186 | 0.02 |
| GATA3 | PTEN | 0.006506 | 0.02 |
| MAP3K1 | KMT2C | 0.00815 | 0.02 |
| BRAF | KMT2C | 0.009522 | 0.02 |
| KMT2D | ERBB4 | 0.013999 | 0.02 |
| TBX3 | CBFB | 0.019772 | 0.03 |
| PIK3R1 | TBX3 | 0.020678 | 0.03 |
| RB1 | MAP2K4 | 0.020733 | 0.03 |
| ARID1A | CBFB | 0.021214 | 0.03 |
| KMT2C | CBFB | 0.021214 | 0.03 |
| MAP3K1 | CBFB | 0.021214 | 0.03 |
| ARID1A | NCOR1 | 0.021986 | 0.03 |
| TP53 | NOTCH2 | 0.022565 | 0.03 |
| TP53 | RB1 | 0.023603 | 0.03 |
| NCOR1 | TP53 | 0.024393 | 0.04 |
| MAP2K4 | EGFR | 0.027742 | 0.04 |
| KMT2C | NOTCH4 | 0.028483 | 0.04 |
| CDH1 | CHEK2 | 0.029262 | 0.04 |
| RB1 | ERBB2 | 0.031176 | 0.04 |
| CDH1 | ARID1A | 0.033809 | 0.04 |
| CDH1 | KMT2C | 0.033809 | 0.04 |
| TBX3 | TP53 | 0.034029 | 0.043 |
| ERBB2 | EGFR | 0.037353 | 0.043 |
| ERBB4 | EGFR | 0.037417 | 0.043 |
| CHEK2 | CBFB | 0.038009 | 0.043 |
| RB1 | NCOR1 | 0.038642 | 0.043 |
| ESR1 | CDH1 | 0.03948 | 0.05 |
| BRCA2 | BRAF | 0.046062 | 0.05 |
| ERBB4 | BRCA1 | 0.047357 | 0.05 |
| PIK3R1 | FOXA1 | 0.047357 |  |

**Supplementary Table 3: Hazard ratios and confidence intervals for TILs samples**

| **Sample type** | **Subtype** | **Number of samples with TILs scored** | **Hazard ratio (confidence intervals)** | **p-value** |
| --- | --- | --- | --- | --- |
| Primary^1^ | All | 115 | 1.18 (0.76-1.84) | 0.4 |
|  | ER+ | 75 | 1.2 (0.60-2.30) | 0.5 |
|  | HER2+ | 10 | 0.70 (0.16-2.79) | 0.6 |
|  | TNBC | 30 | 2.40 (1.05-5.58) | 0.03 |
| Metastatic | All | 105 | 1.04 (0.58-1.86) | 0.8 |
|  | ER+ | 76 | 1.60 (0.70-3.90) | 0.2 |
|  | HER2+ | 9 | 0.30 (0.03-3.96) | 0.3 |
|  | TNBC | 20 | 1.20 (0.40-3.50) | 0.7 |
| (1) TILs significance in primary samples is calculated using the disease free interval between primary and metastatic disease. (2) TILs significance in metastatic disease is calculated from time of metastatic diagnosis to death or follow-up. | | | | |
